# A novel micropropagation of *Lycium ruthenicum* and epigenetic fidelity assessment of three types of micropropagated plants *in vitro* and *ex vitro*

**DOI:** 10.1371/journal.pone.0247666

**Published:** 2021-02-23

**Authors:** Yue Gao, Qin-Mei Wang, Qinxia An, Jianguo Cui, Yongbin Zhou, Xinyu Qi, Lijie Zhang, Lujia Li

**Affiliations:** Key Laboratory of Forest Tree Genetics, Breeding and Cultivation of Liaoning Province, College of Forestry, Shenyang Agricultural University, Shenyang, Liaoning, China; National University of Kaohsiung, TAIWAN

## Abstract

*Lycium ruthenicum* is an excellent eco-economic shrub. Numerous researches have been conducted for the function of its fruits but scarcely focused on the somaclonal variation and DNA methylation. An efficient micropropagation protocol from leaves and stems of *L*. *ruthenicum* was developed in this study, in which not only the leaf explants but also the stem explants of *L*. *ruthenicum* were dedifferentiated and produced adventitious buds/multiple shoots on one type of medium. Notably, the efficient indirect organogenesis of stem explants was independent of exogenous auxin, which is contrary to the common conclusion that induction and proliferation of calli is dependent on exogenous auxin. We proposed that sucrose supply might be the crucial regulator of stem callus induction and proliferation of *L*. *ruthenicum*. Furthermore, results of methylation-sensitive amplified polymorphism (MSAP) showed that DNA methylation somaclonal variation (MSV) of CNG decreased but that of CG increased after acclimatization. Three types of micropropagated plants (from leaf calli, stem calli and axillary buds) were epigenetically diverged more from each other after acclimatization and the *ex vitro* micropropagated plants should be selected to determine the fidelity. In summary, plants micropropagated from axillary buds and leaves of *L*. *ruthenicum* was more fidelity and might be suitable for preservation and propagation of elite germplasm. Also, leaf explants should be used in transformation. Meanwhile, plants from stem calli showed the highest MSV and might be used in somaclonal variation breeding. Moreover, one MSV hotspot was found based on biological replicates. The study not only provided foundations for molecular breeding, somaclonal variation breeding, preservation and propagation of elite germplasm, but also offered clues for further revealing novel mechanisms of both stem-explant dedifferentiation and MSV of *L*. *ruthenicum*.

## Introduction

*Lycium ruthenicum*, belonging to the Solanaceae family, which inhabits northwestern China [1], is a perennial desert pioneer shrub with saline-alkali tolerance, drought resistance, wear tolerance and cold resistance [2–6]. It was also reported to have important medicinal and health-protection values [7]. Due to the important eco-economic values of *L*. *ruthenicum*, a large number of reports on medicinal and health components of its fruits have emerged since 2013 and these reports revealed that the components of the black berries have the functions of anti-radiation [8], regulating intestinal microbiota [9], antioxidant [10], cancer prevention [11], anti-fatigue [12], immuno-enhancement [13, 14], anti-aging [15], delaying the onset and progress of neurodegenerative diseases associated with oxidative stress [16], neuroprotective effect against oxygen-glucose deprivation/reoxygenation-induced neuronal injury in rat primary cortical neurons [17] and so on [18]. In summary, *L*. *ruthenicum* is an excellent eco-economic shrub and worthy of further development, utilization and research. Due to the self-incompatibility of *L*. *ruthenicum* [19], it is difficult to maintain the characters of its parents and produce true-to-type progenies through seed propagation. However, *in vitro* micropropagation may theoretically produce many true-to-type plants. Here we developed an efficient *in vitro* micropropagation protocol from leaves and stems of *L*. *ruthenicum*, also some special characters in auxin demand were found in *L*. *ruthenicum*.

It was reported that DNA methylation patterns are highly variable among various micropropagated plants and between explant donors and micropropagated plants [20–22]. DNA methylation changes arised by micropropagation belong to somaclonal variation (SV) and can also affect phenotype [23]. Moreover, DNA methylation variation is likely to be the leading factor for genetic variation [24]. Thus, we investigated the DNA methylation alterations in *L*. *ruthenicum* plants derived from leaf calli, stem calli and axillary buds in this study. The SV can be used in strain improvement during plant breeding [25, 26], but is undesirable for both long-term genotype preservation and propagation of excellent variety [27]. Some DNA methylation SV (MSV) was heritable via self-pollination of primary regenerants [28], but much DNA MSV in response to the *in vitro* environment cannot be transmitted through meiosis and even mitosis [21]. However, whether the MSV decreases after transplanting is yet to be investigated. Are DNA methylation changes of donor plants in response to acclimatization similar to those of micropropagated plants? Which type, *in vitro* or *ex vitro* micropropagated plants, should be selected to determine the fidelity? Which type of micropropagated plant is more suitable for SV breeding? Which is suitable for germplasm conservation, transformation and propagation of excellent variety? Is there MSV hotspot for *L*. *ruthenicum*? Is the MSV hotspot of plants from calli the same as that of plants from axillary buds? In order to address the questions above, both *in vitro* and *ex vitro* donors & micropropagated plants of *L*. *ruthenicum* were compared in the study. The findings in this study not only provided foundations for molecular breeding, SV breeding, preservation and propagation of excellent germplasm, but also offered clues for further revealing novel mechanisms of both MSV and stem-explant indirect organogenesis of *L*. *ruthenicum*.

## Materials and methods

### Plant materials

No permits were required for the research. The seeds used in the study were collected from the experimental field of our university (Shenyang Agricultural University). Mature seeds of *L*. *ruthenicum* were collected from two plants (PlantD and PlantG) and planted in Shenyang of China (41° 49’ 25” N; 123 ° 34’ 10” E, 60 m above sea level). The seeds were decontaminated with 75% (v/v) alcohol for 30 s, and a 0.1% (w/v) mercuric chloride solution for 2 min, and then rinsed 4 times with sterile distilled water [29]. The sterile seeds were horizontally inoculated on half-strength Murashige and Skoog (1/2 MS) medium [30] without any plant growth regulator (PGR). The 1/2 MS medium was supplemented with 2.0% (w/v) sucrose and 0.50% (w/v) agar (Jinan Zhongtian Plant Tissue culture Center), adjusted to pH 5.8 with KOH prior to autoclaving at 121 °C for 15 min. The inoculated seeds were cultured in dark until they germinated, thereafter the germinated seeds were cultured under 48 μmol m^-2^s^-1^ light provided by LED fluorescent lamps at a photoperiod of 12 h. The temperature is 25±2 °C throughout the course [31]. At 45 days after inoculation, two healthy *in vitro* seedlings of PlantD and G were selected as explant donors. For convenience, the two donors *in vitro* were renamed as *inDdonor* (a seedling from PlantD) and *inGdonor* (a seedling from PlantG). After transplanting, the two donors were renamed as *exDdonor* and *exGdonor*, respectively. Notably, genetic background of the two donors is not identical because they were seedlings from different *L*. *ruthenicum* which shows self-incompatibility.

### Callus, adventitious bud and multiple shoot induction

**Fig 1:**
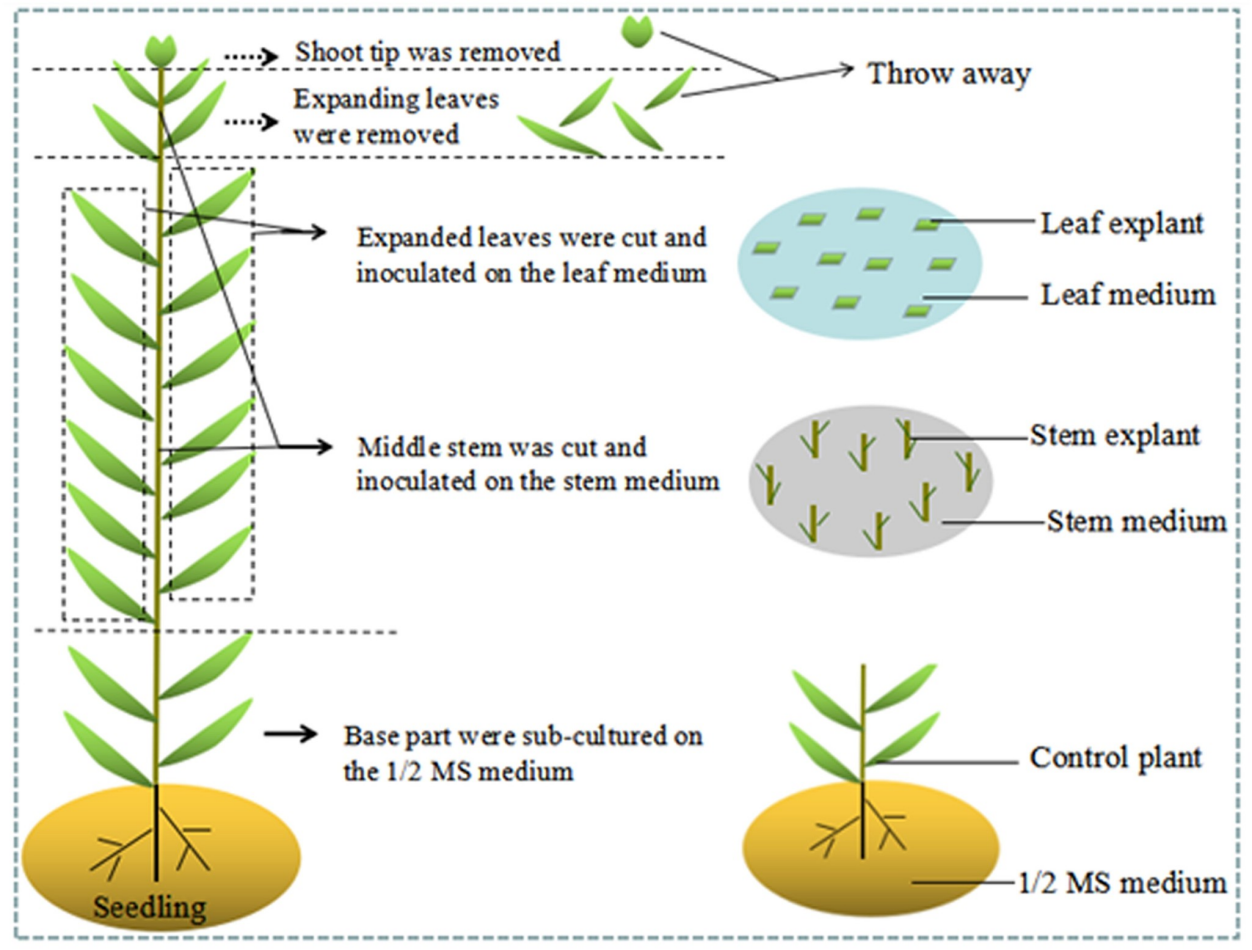
Process used to obtain the leaf and stem explants of *L*. *ruthenicum*.

Expanded leaves of *inDdonor* and *inGdonor* were cut perpendicularly to their main vein into explants about 0.5–0.8 cm, and then inoculated with the abaxial side upwards in flasks with the leaf medium (Fig 1). The leaf medium was Murashige and Skoog (MS) medium [30] supplemented with 4% (w/v) sucrose, 0.50% (w/v) agar, 0.89 μM 6-benzyladenine (6-BA) and 0.54 μM a-naphthaleneacetic acid (NAA). The shoot tips of *inDdonor* and *inGdonor* were removed, the middle stems without leaves were cut into explants with two leaf axils, the remaining base parts of stems with roots and leaves were sub-cultured on the forgoing 1/2 MS medium (Fig 1). The new shoots of the remaining base parts could also be used as leaf and stem explants. The stem explants were inoculated in flasks with the stem medium. It is worth noting that the lower leaf axils of stem explants should touch the stem medium (Fig 1). The stem medium was MS medium supplemented with 4% (w/v) sucrose, 0.50% (w/v) agar and 0.44 μM 6-BA. The concentrations of 6-BA and NAA were chosen in our stem or leaf medium because they result in better micropropagation than other PGR concentrations in our previous experiences. All the inoculated stem and leaf explants were cultured at a photoperiod of 12 h under 48 μmol m^-2^ s^-1^ light provided by LED fluorescent lamps. Prior to autoclaving at 121 °C for 15 min, the PGRs of the leaf and stem medium were added and then the pH of the leaf and stem medium was adjusted to 5.8 with KOH. The temperature (25±2 °C) was maintained throughout the course of *in vitro* culture. Moreover, all the cultures were sub-cultured on the same media every 45 days.

### Rooting and acclimatization of plantlets

When the regenerated shoots were about 2 cm high, they were cut from the leaf-derived calli and transplanted onto the forgoing 1/2 MS medium without any PGR for root induction. Meanwhile, when the shoots from both axillary buds and calli of stem explants were at least 2 cm high, they were cut and inoculated on the forgoing 1/2 MS medium for rooting [31]. The conditions of light intensity, photoperiod and temperature for rooting were identical to those of callus induction. *In vitro* rooted plantlets were acclimated under natural sunlight until both leaves were dark green and stems were no longer tender. Thereafter, the *in vitro* stronger plantlets were transferred into sterilized substrate with the mixture of humus and sphagnum moss (1:1) and the pot was covered with plastic wrap with holes for at least 10 days in a growth room at 25 ± 5 °C under indirect light. Moreover, the matrix were sterilized at 121 °C for 60 min, and the plantlets without medium were immersed in 0.33% (m/v) carbendazim turbid liquid for 5 min before transplanting. After removing the plastic wrap film, the transplanted *ex vitro* plantlets were originally acclimated under indirect sunlight for 10–20 days and then were exposed to direct sunlight.

### DNA isolation and quantification

**Fig 2:**
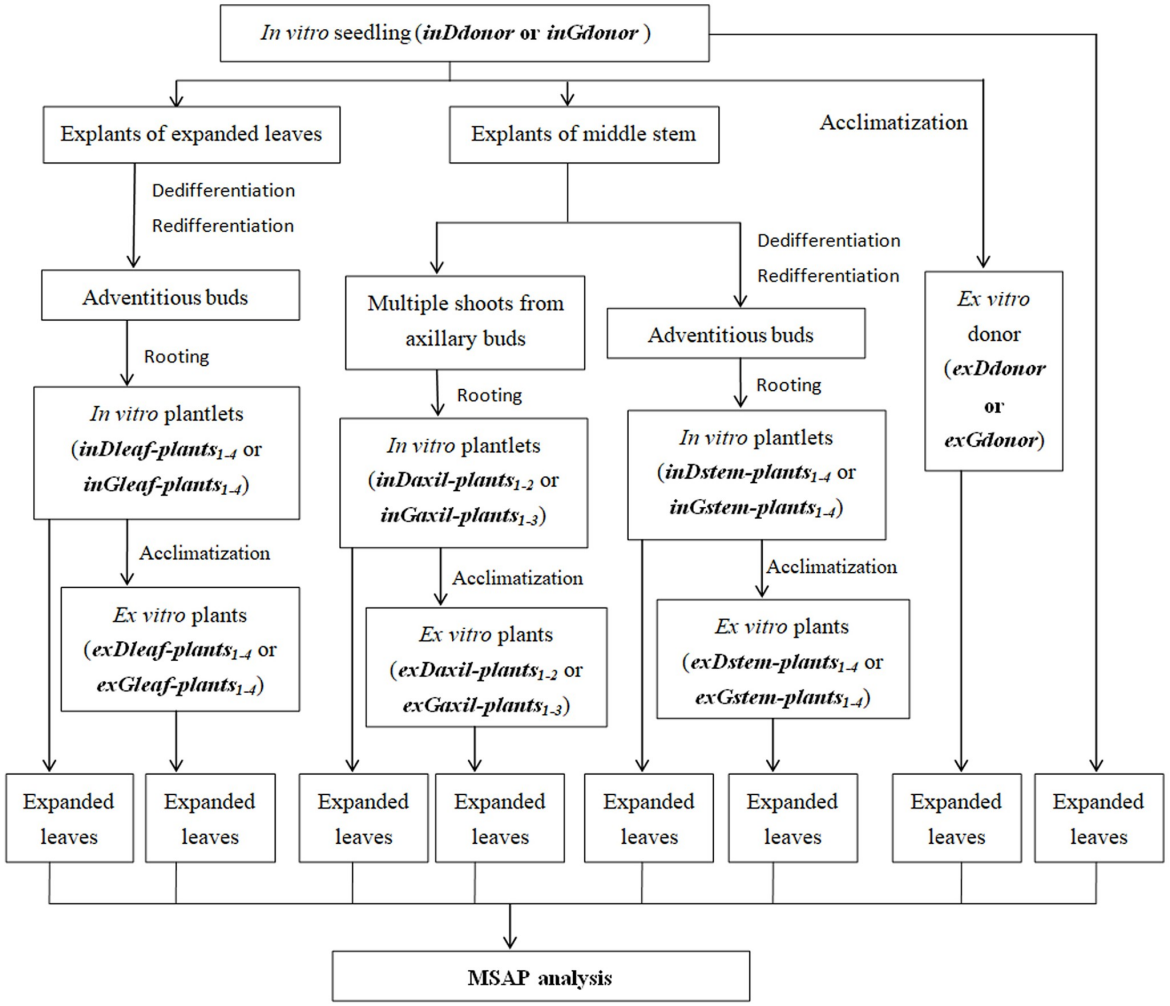
Process used to obtain *L*. *ruthenicum* samples of D and G groups used in MSAP analysis. The abbreviations of plants are shown in brackets. Subscripts indicated the number of the plants used in the analysis.

Genomic DNA was extracted from the expanded leaves of the two donor plants *in vitro* and *ex vitro*, from expanded leaves of both *in vitro* and *ex vitro* micropropagated plants derived from leaf calli, stem calli and axillary buds using a small-scale DNA isolation method (NuClean Plant Genomic DNA Kit-CVVBIO) according to the manufacturers’ instructions. Notably, the expanded leaves from the new shoots of the transplanted plants were used for DNA extraction in order to exclude the non-heritable DNA MSV in response to the *in vitro* environment. Two replicate extractions from all the samples above were performed. DNA purity, integrity and concentration were assessed by the method mentioned in our previous report [22]. Fig 2 shows the processes used to obtain the samples used in MSAP analysis and the sample numbers. Meanwhile, for convenience, the micropropagated plant samples were renamed as follows (Fig 2): *inDaxil-plant*_*1-2*_ (*in vitro* plants from axillary buds of *Ddonor*), *inDstem-plant*_*1-4*_ (*in vitro* plants from stem calli of *Ddonor*), *inDleaf-plant*_*1-4*_ (*in vitro* plants from leaf calli of *Ddonor*), *exDaxil-plant*_*1-2*_ (transplanted plants from axillary buds of *Ddonor*), *exDstem-plant*_*1-4*_ (transplanted plants from stem calli of *Ddonor*), *exDleaf-plant*_*1-4*_ (transplanted plants from leaf calli of *Ddonor*), *inGaxil-plant*_*1-3*_ (*in vitro* plants from axillary buds of *Gdonor*), *inGstem-plant*_*1-4*_ (*in vitro* plants from stem calli of *Gdonor*), *inGleaf-plant*_*1-4*_ (*in vitro* plants from leaf calli of *Gdonor*), *exGaxil-plant*_*1-3*_ (transplanted plants from axillary buds of *Gdonor*), *exGstem-plant*_*1-4*_ (transplanted plants from stem calli of *Gdonor*), *exGleaf-plant*_*1-4*_ (transplanted plants from leaf calli of *Gdonor*).

### MSAP analysis

For the purpose of (a) detecting the differences of MSV among *in vitro* plants from leaf calli, axillary buds and stem calli and among *ex vitro* plants from leaf calli, axillary buds and stem calli, (b) revealing the differences of MSV between the micropropagated plants *in vitro* and *ex vitro*, and (c) finding the micropropagated plant-specific MSAP markers, the MSAP method was employed to assess cytosine methylation differences in D and G groups (Fig 2). All the adapters and primers used in MSAP were custom synthesized from GENEWIZ (Hangzhou, China). The T4 ligase and restriction enzymes *Eco*RI, *Hap*II and *Msp*I were purchased from New England Biolabs Inc. The MSAP method using capillary electrophoresis (CE) of our previous report [29] was followed except for the selective primer combinations (S1 Table). Only *Eco*RI + 3 primers were 5’-end-labeled using 6-carboxy-2’, 4, 4’, 5’, 7, 7’-hexachlorofluorescein (HEX), 6-carboxyfluorescein (FAM) or TAMARA (GENEWIZ, Suzhou, China) to allow product detection during CE on an ABI 3730XL (S1 Table) [29]. For pre-amplification and selective amplification reaction, our previous PCR thermal cycler conditions were used [22].

### Data analysis

All the data of micropropagation were subjected to statistical analysis using paired sample t-test (2-tailed, P<0.05). The scored MSAP bands were transformed into a binary character for the absence (0) or presence (1). All the binary data of MSAP were generated by software GeneMarker V2.2.0 (SoftGenetics, USA). The levels of cytosine (CCGG sites) methylation and locus-specific methylation differences among samples within D or G group were subjected to statistical analysis using one sample t-test (2-tailed, P<0.01 and 0.05) and one-way ANOVA (2-tailed, P<0.05) by software SPSS ver. 20.0 (IBM Co., Armonk, NY, USA) [29]. Meanwhile, independent-sample t-test of SPSS ver. 20.0 was used to compare the *in vitro* and *ex vitro* locus-specific MSV of the same micro-propagated plants. Moreover, the principal coordinate analysis (PCA) and UPGMA cluster analysis of MSAP profiles were carried out by software MVSP ver. 3.2 (Kovach Computing Services, Wales, U.K.) [32]. The specific MSAP markers were determined as follows: (1) all MSAP sites that showed a monomorphic pattern or a ‘Suspected’ by GeneMarker V2.2.0 in only one sample were excluded from the binary data matrices [33]; (2) the remaining binary data matrices were transformed into quaternion matrices (00→0, 01→1, 10→2, 11→3) by excel data processing; (3) the micro-propagated- or *ex vitro* plant-specific MSAP markers were found and determined [34].

## Results

### Leaf explant produced both calli and adventitious buds on the same medium

**Fig 3:**
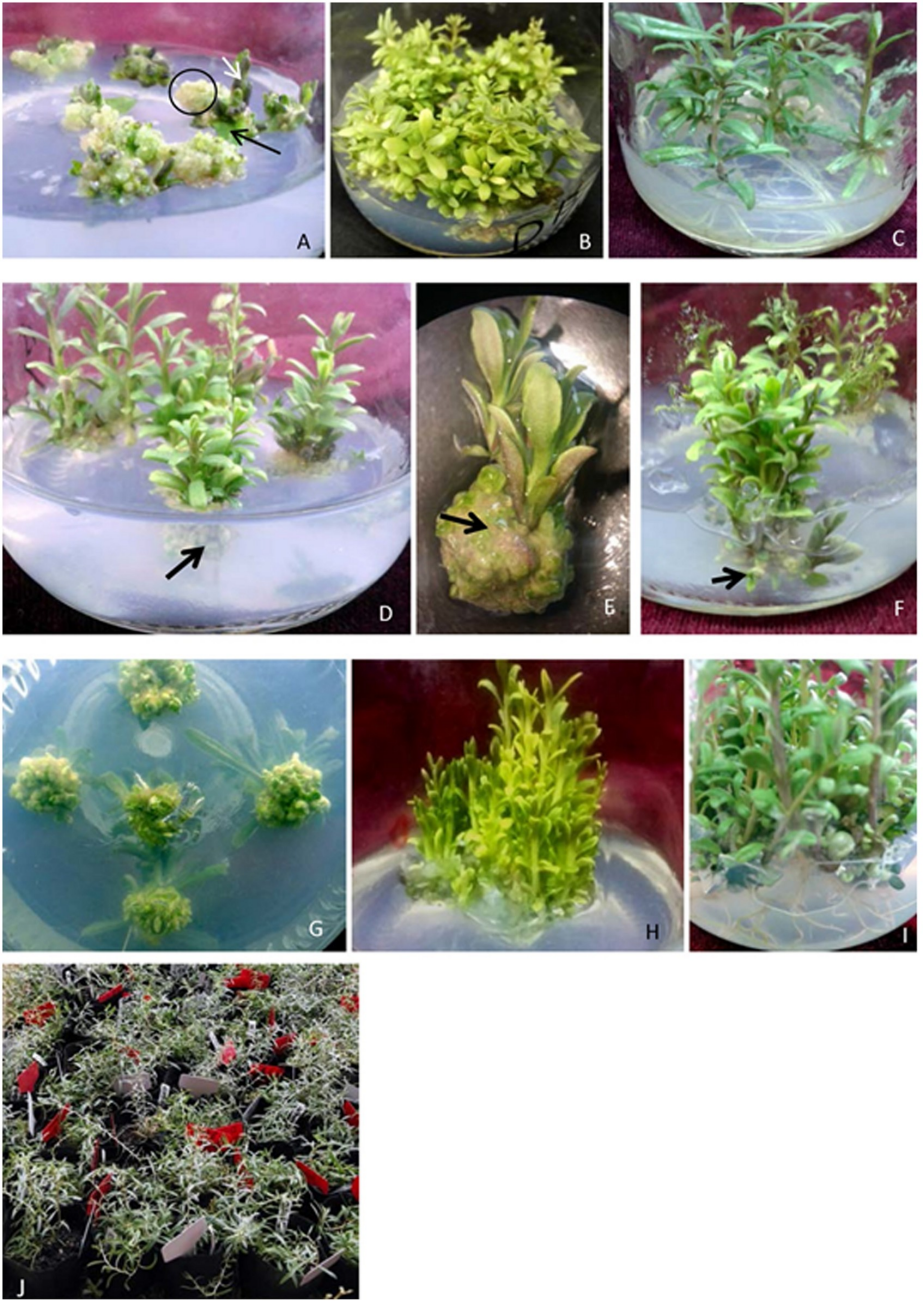
Plant regeneration of *L*. *ruthenium* from leaves and stems. (A) Callus (circle) and adventitious buds (white arrow) derived from leaf explant (black arrow); (B) Rosette shoots from leaf callus; (C) The rooted plantlets from leaf explants; (D) Multiple shoots from axillary bud and nodular callus (black arrow) from cross section of stem explant; (E) The nodular callus (arrow) was magnified; (F) (G) Adventitious buds (arrow in F) from nodular callus of stem explant; (H) All the shoots from a single stem explant; (I) The rooted plantlets from stem explant; (J) Plantlets after acclimatization.

In the leaf medium, not only the leaf explants from the two donors (*inGdonor* and *inDdonor*) produced calli but also adventitious buds were regenerated from the calli (Fig 3A). After 30 days of culture on the leaf medium, the leaf explants of two donors showed similar efficiency for callus induction; however, the percentage of callus producing adventitious buds was significantly lower for materials from *inGdonor* than that from *inDdonor* (Table 1). This indicated that adventitious buds were regenerated in a genetic background-dependent pattern. Moreover, after 60 days of culture on the leaf medium, shoots produced by the calli could reach the height of 2–3 cm (Fig 3B) and be used for *in vitro* root induction.

**Table 1:** Effect of genetic background on callus, multiple shoot, adventitious bud and root induction in *L*. *ruthenicum*.

Donor plant	*inDdonor*	*inGdonor*
**Frequency of callus development from leaves (%)**	99.21±0.79^a^	100.00±0.00^a^
**Percentage of leaf callus producing adventitious buds (%)**	86.26 ±8.98^a^	30.11±8.45^b^
**Percentage of stem explant producing multiple shoots (%)**	96.67±3.33^a^	86.41 ±2.29^b^
**Percentage of stem explant producing calli with adventitious buds (%)**	71.90±4.54^a^	47.07±3.8 ^b^
**Rooting rate of shoots from leaf explants (%)**	59.47±1.58 ^c^	88.02 ±1.85^ab^
**Rooting rate of shoots from stem explants (%)**	81.43 ±2.18^b^	93.87±0.17 ^a^

Each value represents mean ± SE of three replicates. Data within lines labeled with different letters are significantly different at the 0.05 level by t-test.

### Stem explants produced two types of shoots on auxin-free medium

On the auxin-free MS medium supplemented with 0.44 μM 6-BA (stem medium), the stem explants of *inGdonor* and *inDdonor* produced not only multiple shoots from axillary buds but also nodular calli from cross section enwrapped by the medium (Fig 3D and 3E). On the same stem medium, adventitious buds were regenerated from the nodular calli soon (Fig 3F and 3G) and grew rapidly (Fig 3H). After 30 days of culture on the stem medium, both the percentage of D stem explants producing multiple shoots and producing calli with adventitious buds were significantly higher than that of G stem explants (Table 1). This suggested that both the axillary bud germination and regeneration of *L*. *ruthenicum* stem explants were affected by the genetic background.

### Rooting and acclimatization of plantlets

A total of 59.47–93.87% shoots rooted *in vitro* on the 40th day after being transferred onto the 1/2 MS medium without any PGR (Table 1, Fig 3C and 3I). Rooting rate of shoots from stem explants was higher than that from leaf explants of the same donor (Table 1). Meanwhile, rooting rate of G clones was significantly higher than that of D clones (Table 1). Totally, both genetic background and explant type affected the rooting capability of *L*. *ruthenicum* shoots. The transplanting survival rate of *L*. *ruthenicum* plantlets was up to 95.65% by using the acclimatization protocol above (Fig 3J).

### Selection of suitable primer pairs for MSAP analysis

Fourteen primer combinations were selected (S1 Table) based on the criteria of our previous report [22]. Using the 14 primer pairs, we scored 1,751 and 1,743 reproducible bands from D and G group, respectively (Tables 2 and 3). Within D group, 1,458 (83.27%) of the 1,751 bands were polymorphic in either double digestion. Meanwhile, the total methylation polymorphism frequency of G group was 86.69%. The 14 primer combinations all resulted in polymorphic bands within D or G group.

**Table 2:** Cytosine methylation level of *L*. *ruthenicum* D group based on MSAP analysis using 14 primer pairs.

Donor plant and regenerants	Total bands	Unmethylated CCGG sites (%)	Methylated CCGG sites			
			CG (%)	CNG (%)	CG & CNG (%)	Total (%)
***inDdonor***	1,751	759 (43.35)	397 (22.67)	163 (9.31)	432 (24.67)	992 (56.65)
***inDaxil-plants***	1,751	784 (44.77)^a^	235.50 (13.45)^b^	179 (10.22)^bc^**	552.50 (31.55)^ab^*	967 (55.23)^a^
***inDstem-plants***	1,751	756.25 (43.19)^a^	278.50 (15.19)^b^**	163.50 (9.34)^c^	552.75 (31.57)^a^**	994.75 (56.81)^a^
***inDleaf-plants***	1,751	767.25 (43.82)^a^	289.75 (16.55)^ab^*	163.25 (9.32)^c^	530.75 (30.31)^ab^*	983.75 (56.18)^a^
**Mean of *in vito***	1,751	765.55 (43.72)	285.55 (16.31)	166.18 (9.49)	533.73 (30.48)	985.45 (56.28)
***exDdonor***	1,751	788 (45.00)	287 (16.39)	242 (13.82)	434 (24.79)	963 (55.00)
***exDaxil-plants***	1,751	780.50 (44.57)^a^	318.50 (18.19)^ab^	215.50 (12.31)^ab^	436.50 (24.93)^c^	970.50 (55.43)^a^
***exDstem-plants***	1,751	756.75 (43.22)^a^	350.75 (20.03)^a^	189.50 (10.82)^bc^*	454 (25.93)^c^	994.25 (56.78)^a^
***exDleaf-plants***	1,751	770.25 (43.99)^a^	290.75 (16.60)^ab^	235.75 (13.46)^a^	454 (25.94)^c^	980.75 (56.01)^a^
**Mean of *ex vitro***	1,751	768.82 (43.91)	317.27 (18.12)	215.82 (12.33)	449.09 (25.65)	982.18 (56.09)

* indicates significant difference (P<0.05) compared to control plant (*inDdonor* or *exDdonor*).

** indicates extremely significant difference (P<0.01) compared to control plant (*inDdonor* or *exDdonor*).

Data within columns labeled with different letters are significantly different at the 0.05 level by LSD of one-way ANOVA.

**Table 3:** Cytosine methylation level of *L*. *ruthenicum* G group based on MSAP analysis using 14 primer pairs.

Donor plant and regenerants	Total bands	Unmethylated CCGG sites (%)	Methylated CCGG sites			
			CG (%)	CNG (%)	CG & CNG (%)	Total (%)
***inGdonor***	1,743	808 (46.36)	212 (12.16)	275(15.78)	448(25.70)	935 (53.64)
***inGaxil-plants***	1,743	788 (45.21)^abc^	274.33 (15.74)^b^	247.33 (14.19)^a^	433.33 (24.86)^ab^	955 (54.79)^ab^
***inGstem-plants***	1,743	764.25 (43.85)^abc^*	253.25 (14.53)^b^**	222 (12.74)^a^	503.50 (28.89)^a^*	978.75 (56.15)^ab^*
***inGleaf-plants***	1,743	803.50 (46.10)^ab^	259 (14.86)^b^**	255 (14.63)^a^*	425.50 (24.41)^ab^	939.50 (53.90)^b^
**Mean of *in vitro***	1,743	786.92 (45.15)	257 (14.74)	243.75 (13.98)	455.33 (26.12)	956.08 (54.85)
***exGdonor***	1,743	791 (45.38)	291 (16.70)	283 (16.24)	378 (21.69)	952 (54.62)
***exGaxil-plants***	1,743	815 (46.76)^a^	266 (15.26)^b^	242.33 (13.90)^a^	419.67 (24.08)^ab^	928 (53.24)^b^
***exGstem-plants***	1,743	722.50 (41.45)^c^	361.25 (20.73)^a^	217.25 (12.46)^a^*	442 (25.36)^ab^	1020.50 (58.55)^a^
***exGleaf-plants***	1,743	763.57 (43.82)^abc^	345.75 (19.84)^a^*	219.75 (12.61)^a^*	413.75 (23.74)^b^	979.25 (56.18)^ab^
**Mean of *ex vitro***	1,743	765.08 (43.89)	326.42 (18.73)	229.83 (13.19)	421.67 (24.19)	977.92 (56.11)

* indicates significant difference (P<0.05) compared to control plant (*inGdonor* or *exGdonor*).

** indicates extremely significant difference (P<0.01) compared to control plant (*inGdonor* or *exGdonor*).

Data within columns labeled with different letters are significantly different at the 0.05 level by LSD of one-way ANOVA.

### Changes in cytosine methylation level occurred between donors and micropropagated plants of *in vitro* and *ex vitro*

Of the CCGG sites assessed in plants *in vitro* and *ex vitro*, 53.64–56.81% and 53.24–58.55% are methylated, respectively (Tables 2 and 3). Moreover, compared with *ex vitro* plants, the average internal cytosine (CG) methylation levels in *in vitro* plants of the two groups are all lower (Tables 2 and 3). Compared with the corresponding *in vitro* donors of the two groups, all the *in vitro* plantlets regenerated from stem callus showed two types of significant alterations in three types of detectable cytosine methylation levels (CG, CNG and CG & CNG, Tables 2 and 3). However, there was only one type of significant difference between the *ex vitro* donors and the plants from stem calli within each group after the transplant (Tables 2 and 3). Interestingly, for plants regenerated from stem calli, the types of significant alterations *ex vitro* were different from those of *in vitro* within each of the two groups (Tables 2 and 3). This revealed that after acclimatization, the significant alterations *in vitro* might be erased but the *in vitro* non-significant alterations became significant. Compared with *inDdonor*, the alterations of methylated CG and CG & CNG in plants from D leaves are statistically significant; however, after acclimatization the alterations were never statistically significant (Table 2). Nevertheless, that was not the same as plants from leaf calli of G group, whose significant different levels *in vitro* (CG and CNG) still significant after transplanting (Table 3). Compared with donors, only *in vitro* D plants from axillary buds showed significant alterations in methylated CNG and CG & CNG (Table 2). These results indicated that methylation level SV of *L*. *ruthenicum* micropropagated plants was mainly reduced after acclimatization but some level SV in plants from stem calli increased after acclimatization.

### Locus-specific methylation alterations occurred both *in vitro* and *ex vitro*

Compared with the *in vitro* donor plants, all the patterns of locus-specific methylation alterations in three types of micro-propagated plants *in vitro* were statistically significant at 0.01 levels (Table 4). Broad DNA methylation changes occurred during *in vitro* cultures of *L*. *ruthenicum* and there were some differences among the three types of plants. The average levels of CG hypomethylation (CG Hypo), CG & CNG hypomethylation (Both Hypo) and total methylation changes (Total Hyper + Total Hypo) in plants from stem calli were all the lowest and the counterparts in plants from leaf-calli, however, were all the highest (Table 4). To sum up, *in vitro L*. *ruthenicum* plants arranged in increasing order according to levels of total locus-specific methylation alterations relative to the *in vitro* donors are as follows: plants from stem calli, plants from axillary buds and plants from leaf calli. Moreover, for each types of the micropropagated plants *in vitro*, CG Hyper>CG Hypo; CNG Hyper>CNG Hypo; Total Hyper>Total Hypo (Table 4).

**Table 4:** Changes in cytosine methylation pattern in the *in vitro* plants from leaf calli, stem calli and axillary buds compared with the corresponding *in vitro* donors of *L*. *ruthenicum*.

Comparison within two groups	Patterns [frequencies (%)]								
	CG Hyper	CG Hypo	CNG Hyper	CNG Hypo	Both Hyper	Both Hypo	Total Hyper	Total Hypo	Total
***inDaxil-plants* vs. *inDdonor***	3.28	4.31	17.30	10.19	1.46	1.77	22.04	16.28	38.32
***inGaxil-plants* vs. *inGdonor***	8.53	6.64	8.53	9.05	1.57	1.36	18.63	17.04	35.67
**Mean1**	5.91^a^**	5.47^ab^**	12.92^a^**	9.62^a^**	1.51^a^**	1.56^a^**	20.34^a^**	16.66^a^**	36.99^ab^**
***inDstem-plants* vs. *inDdonor***	4.51	4.43	16.30	10.71	1.67	0.99	22.49	16.12	38.61
***inGstem-plants* vs. *inGdonor***	10.01	4.93	10.01	5.85	1.33	1.08	21.36	11.86	33.22
**Mean2**	7.26^a^**	4.68^b^**	13.16^a^**	8.28^a^**	1.50^a^**	1.03^b^**	21.92^a^**	13.99^b^**	35.91^b^ **
***inDleaf-plants* vs. *inDdonor***	4.83	5.44	15.72	11.28	2.30	1.63	22.84	18.35	41.19
***inGleaf-plants* vs. *inGdonor***	9.62	8.78	9.62	7.44	1.48	1.59	20.73	17.81	38.54
**Mean3**	7.23^a^**	7.11^a^**	12.67^a^**	9.36^a^**	1.89^a^**	1.61^a^**	21.78^a^ **	18.08^a^**	39.87^a^**

* Difference at 0.05 level by one-sample t-test;

** Difference at 0.01 level by one-sample t-test.

Data within columns labeled with different letters are significantly different at the 0.05 level by LSD of one-way ANOVA.

All the patterns of locus-specific methylation alterations in three types of micropropagated plants *ex vitro* versus the *ex vitro* donor plants were statistically significant with Both Hypo and Both Hyper of plants from axillary buds at 0.05 levels and the others at 0.01 levels (Table 5). After transplanting, the level of CG Hyper in plants from stem calli was significantly higher than that in plants from axillary buds. However, the level of CG Hypo was contrary to that of CG Hyper (Table 5). Unlike *in vitro* plants, the total level of locus-specific methylation alterations in the *ex vitro* plants from stem calli was the highest but there was no statistical significance. Just like that *in vitro*, for each type of the micropropagated plants *ex vitro*, the level of hypermethylation was higher than that of hypomethylation (CG Hyper>CG Hypo, CNG Hyper<CNG Hypo, Both Hyper>Both Hypo, Total Hyper>Total Hypo).

**Table 5:** Changes in cytosine methylation pattern in the *ex vitro* plants regenerated from leaf calli and stem calli, and *ex vitro* plantlets derived from axillary buds compared with the corresponding *ex vitro* donors of *L*. *ruthenicum*.

Comparison within two groups	Patterns [frequencies (%)]								
	CG Hyper	CG Hypo	CNG Hyper	CNG Hypo	Both Hyper	Both Hypo	Total Hyper	Total Hypo	Total
***exDaxil-plants* vs. *exDdonor***	9.17	7.62	6.23	7.20	1.17	1.17	16.56	15.99	32.55
***exGaxil-plants* vs. *exGdonor***	7.48	8.19	6.04	6.39	2.31	1.28	15.83	15.85	31.69
**Mean1**	8.32^b^**	7.90^a^**	6.13^a^**	6.79^a^**	1.74^a^*	1.23^a^*	16.20^a^**	15.92^a^**	32.12^a^**
***exDstem-plants* vs. *exDdonor***	10.14	6.05	6.05	7.70	1.36	1.11	17.55	14.86	32.41
***exGstem-plants* vs. *exGdonor***	11.98	6.47	6.74	7.20	2.71	1.43	21.43	15.10	36.53
**Mean2**	11.06^a^**	6.26^b^**	6.40^a^**	7.45^a^**	2.03^a^**	1.27^a^**	19.49^a^**	14.98^a^**	34.47^a^**
***exDleaf-plants* vs. *exDdonor***	9.51	8.35	6.30	5.60	1.03	0.87	16.83	14.82	31.65
***exGleaf-plants* vs. *exGdonor***	9.67	6.88	6.04	7.07	2.18	1.25	17.89	15.20	33.09
**Mean3**	9.59^ab^**	7.62^ab^**	6.17^a^**	6.33^a^**	1.60^a^**	1.06^a^**	17.36^a^**	15.01^a^**	32.37^a^**

* Difference at 0.05 level by one-sample t-test;

** Difference at 0.01 level by one-sample t-test.

Data within columns labeled with different letters are significantly different at the 0.05 level by LSD of one-way ANOVA.

From the data of Tables 4 and 5 we concluded that internal cytosine MSV of CCGG sites (CG Hyper and CG Hypo) in three types of micropropagated plants increased and that of CNG (external cytosine) decreased after acclimatization (Table 6). Also, the levels of CG Hyper in plants from calli of both leaf and stem were significantly increased (P<0.01) after acclimatization (Table 6). However, after acclimatization the levels of CNG Hyper in all the three types of micropropagated plants were significantly decreased (P<0.01) (Table 6). There were one and two patterns of significant methylation alterations in plants from axillary buds and calli, respectively (Table 6), suggesting that the MSV of plants from two types of calli showed the sharpest changes in response to acclimatization and the smallest changes were in plants from axillary buds. In total, neither methylation level SV or locus-specific MSV of *L*. *ruthenicum* showed a simple reduction after acclimatization because there was not only decrease but also increase, however, the decrease was predominant.

**Table 6:** Locus-specific MSV in *ex vitro* plants from axillary buds, stem calli and leaf calli compared with that of the corresponding *in vitro* plants of *L*. *ruthenicum*.

Patterns	Plants from axillary buds	Plants from stem calli	Plants from leaf calli
**CG Hyper**	↑	↑**	↑**
**CG Hypo**	↑	↑	↑
**CNG Hyper**	↓**	↓**	↓**
**CNG Hypo**	↓	↓	↓

^↑^ Increase,

^↓^ Decrease,

** Significant difference at 0.01 level by independent-sample t-test.

All the patterns of locus-specific methylation alterations in leaves of various micropropagated plants *ex vitro* versus the corresponding *in vitro* leaves of the same plants were found to be statistically significantly. However, the total locus-specific methylation alterations in the two donors (44.24%) were the sharpest (S2 Table), which indicated that donors and micropropagated plants did not show the identical response to acclimatization. For all the three types of micropropagated plants, the rates of CNG Hypo and Total in plants from stem calli were the highest (S2 Table). This might account for why the significant methylation level alterations in plants from stem calli were erased but the novel significant alterations appeared after acclimatization (Tables 2 and 3).

### Epigenetic divergence among all the plants within each group

**Fig 4:**
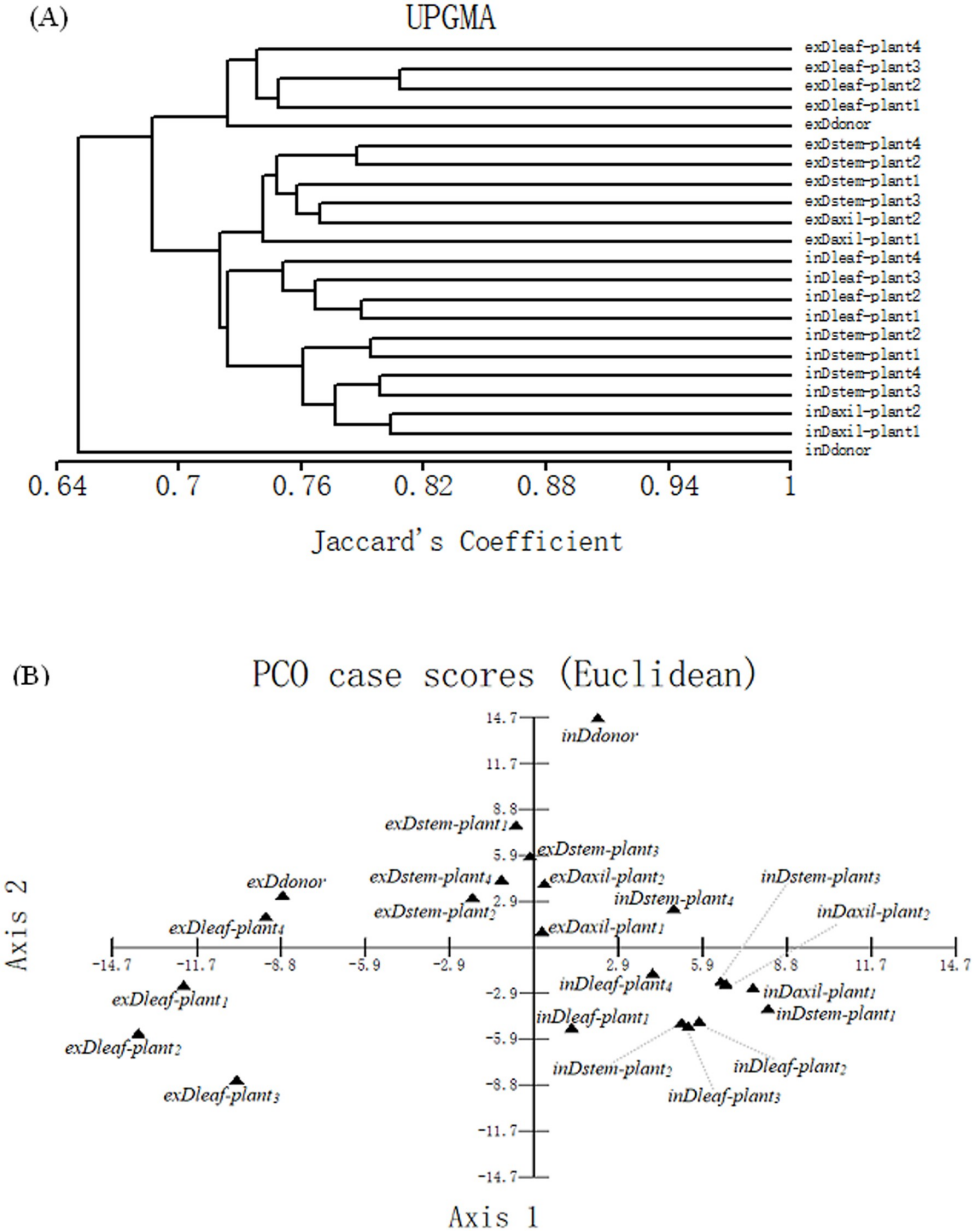
Dendrogram illustrating coefficient similarities among samples in *L*. *ruthenicum* D group by the UPGMA cluster analysis based on the MSAP profiles (A), and associations among the samples in D group revealed by PCA (B).

Cluster analysis of D group based on the MSAP profiles revealed that (a) the *in vitro* and *ex vitro* plants from stem explants were clustered into two separate groups, respectively; (b) all the *in vitro* plants regenerated from leaves were clustered into another group; (c) all the *in vitro* micropropagated plants were diverged more from the *in vitro* donor than from each other but (d) after the acclimatization, the plants from leaf calli were clustered with *ex vitro* donor preferentially (Fig 4A). The PCA further validated the results of cluster analysis (Fig 4B). The above (a)-(c) implied that MSV *in vitro* are not random and are somewhat consistent between three types of *in vitro* micropropagated plants, consistent with the finding that consistent alterations of DNA methylation are induced by tissue culture in maize [28]. Moreover, the above (c)-(d) suggested that it was easier to select the most fidelity micropropagated plants after transplanting.

**Fig 5:**
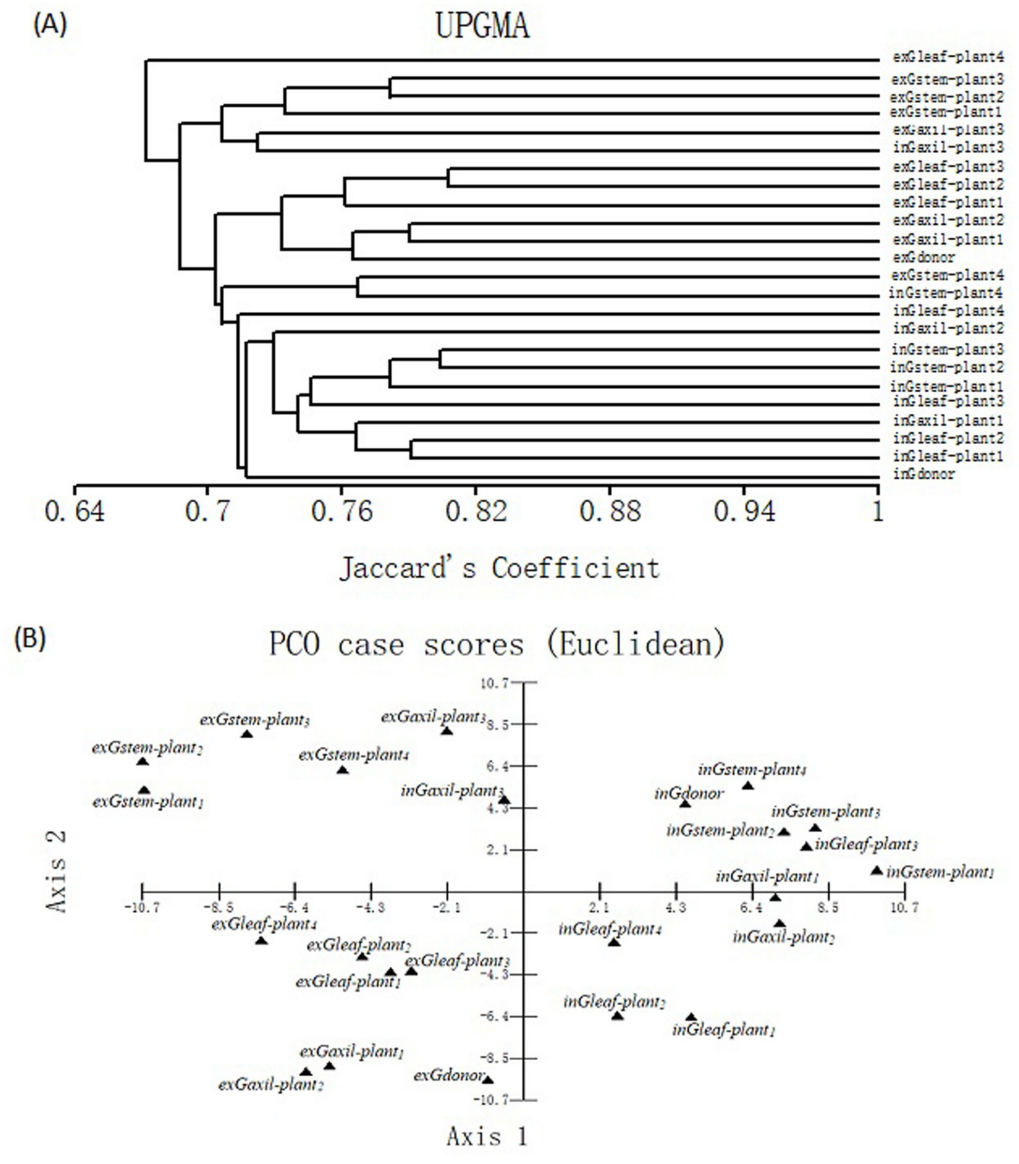
Dendrogram illustrating coefficient similarities among samples in *L*. *ruthenicum* G group by the UPGMA cluster analysis based on the MSAP profiles (A), and associations among the samples in G group revealed by PCA (B).

Both cluster and PCA results of group G and D were similar. However, the special sample *exGstem-plant*_*4*_ was clustered into one group with plants *in vitro*; the special sample *inGaxil-plant*_*3*_ was clustered into one group with plants *ex vitro* (Fig 5A). It was difficult to conclude which type(s) of *in vitro* plants was more similar to the *in vitro* donor. However, after acclimatization, the plants from stem calli exhibited significantly more epigenetic divergence from donor than those from both axillary buds and leaf calli with two plants from axillary buds most fidelity (Fig 5B). It was difficult to select the most fidelity micropropagated plants *in vitro* but easier *ex vitro*.

### Micropropagated plant-specific MSAP sites

Compared with the *in vitro* donors, 14 and two *in vitro* micropropagated plant-specific MSAP sites were found in D and G groups, respectively (S3 and S4 Tables), but none of these were transmitted to the *ex vitro* leaves. This indicated that some main MSV in *L*. *ruthenicum* cannot be transmitted through mitosis. The sites of H4-153 are ‘11’ in all the *in vitro* micropropagated plants of D group and ‘01’ in *inDdonor* (S3 Table). Meanwhile, the site of H4-153 in G group except *inGaxil-plant*_*3*_ (a special sample in cluster) was the same as that in D group, which indicated that methylation modification in site of H4-153 was usually removed during *in vitro* culture, and the site can be regarded as *in vitro* MSV hotspot but the demethylation of H4-153 site is not a necessory event for micropropagation of *L*. *ruthenicum*, because not all the micropropagated plants show the same alteration. Nevertheless, all the *in vitro* plants from calli show the same alteration. Moreover, the majority of the micropropagated plant-specific sites are only observed in one of the two groups, indicating that MSV of *L*. *ruthenicum* is depend on genetic background. Although the significantly locus-specific methylation alterations between the *in vitro* and *ex vitro* leaves of the same plants existed, we did not find any *in vitro* plant-specific MSAP site.

## Discussion

### Micropropagation of *L*. *ruthenicum*

Dedifferentiation and redifferentiation rates of stem explants (Table 1), redifferentiation rates of leaf callus (Table 1), percentage of stem explant producing multiple shoots (Table 1) and rooting rates (Table 1) were all significantly different between the two groups, indicating that stem explant dedifferentiation and redifferentiation, multiple shoots formation from axillary bud, redifferentiation of leaf callus and rooting of *L*. *ruthenicum* were all affected by genetic background.

Calli of *L*. *ruthenicum* stem and leaf could regenerate adventitious buds on the callus induction medium soon. That is to say, shoot regeneration of *L*. *ruthenicum* did not need change the proportion of exogenous auxin and cytokinin, which is contrary to the common accepted view that calli can be induced from explants on the medium supplemented with an optimal concentration of exogenous auxin and cytokinin, and subsequent culture of the calli with high cytokinin/auxin ratio leads to shoot regeneration [35, 36]. Moreover, the roots of *L*. *ruthenicum* were induced without exogenous auxin, which is similar to that of the *Clivia miniata* [31] and *Brassica juncea* var. *Tsatsai* [37] but in contrast to the majority of previous studies which found proper exogenous auxin promotes rooting of *in vitro* shoots [35]. It is commonly accepted that exogenous auxin plays a key role for plant callus induction and proliferation, however, exogenous cytokinin, may play a coordinating role for callus induction and proliferation [35, 38]. In addition, proliferation of habituated *Arabidopsis* callus is dependent on exogenous auxin but not on cytokinin [20]. Furthermore, exogenous auxin-picloram or indolo acetic acid was necessary for callus induction from undifferentiated cambial meristematic cells [39]. Surprisingly, the stem explants of *L*. *ruthenicum* could produce calli without exogenous auxin. Above all, *L*. *ruthenicum* showed abnormal auxin demand during its micropropagation process. Not only did stem explants of *L*. *ruthenicum* produce calli but also *in vitro* shoots of *L*. *ruthenicum* rooted without exogenous auxin. It was proposed that the leaf-to-callus process is not a dedifferentiation process but a transdifferentiation process [36, 40] because many studies in *Arabidopsis* had demonstrated that callus is a group of root meristem tip cells and that callus induction resembles lateral root formation [41–44]. Therefore, we proposed that callus induction from stem cross section enwrapped by medium exceedingly resembles root induction from shoot of *L*. *ruthenicum*. Also, not only the roots but also the forgoing stem callus should be originated from vascular cambium of *L*. *ruthenicum* stem [45–47]. Therefore, the stem callus induction without exogenous auxin in fact is identical to root induction without exogenous auxin. Notably, exogenous auxin is an essential regulator of callus initiation and proliferation in other plants [36] but not in *L*. *ruthenicum* stem of this study. Thus, a novel mechanism of callus initiation and proliferation might exist in *L*. *ruthenicum* and even in other plants. Recent researches have shown that sugar demand, not auxin, is the initial regulator of apical dominance [48, 49]. Moreover, developmental transitions in plants require adequate sugar which acts as sugar signaling and carbon energy-supply [49, 50]. We concluded that sucrose supply maybe the crucial regulator of stem callus initiation and proliferation in *L*. *ruthenicum* because only the lower stem cross section enwrapped by medium with 4% (w/v) sucrose and without auxin could produce callus (Fig 3D) but the upper stem cross section did not. However, further study should be carried out to clarify the hypothesis.

### DNA methylation variation and specific MSAP sites

In this survey, the total cytosine methylation levels in expanded leaves of *L*. *ruthenicum* are 53.64–56.81% *in vitro* and 53.24–58.55% *ex vitro*. The methylation levels are certainly higher than those surveyed in almost all other plants [37, 51–55] but comparable to those in *Clivia miniata* [22]. However, the CG methylation levels in *L*. *ruthenicum* are significantly lower than those in diploid *C*. *miniata* [22] and tetraploid cotton [56]. To the best of our knowledge, these are the first data on DNA methylation of *L*. *ruthenicum*.

The PCA revealed total clear separation between leaves of plants *in vitro* and leaves of the same plants *ex vitro* within D group. The result was similar to a previous report which found clear separation between *in vitro* propagated plants and their field counterparts from cuttings for five *Manihot esculenta* cultivars [57]. Notably, plants compared in Kitimu’s study are different plants cloned by two methods but leaves compared in this study are from the same plants before and after acclimatization. Thus, we can deduce more rigorous conclusion of plasticity in genomes of *L*. *ruthenicum* growing under two different environments.

Initially, we proposed that after acclimatization the MSV should decrease because only the heritable SV existed in the new leaves of transplanted plants. It was supported by the MSV hotspot which was not transmitted to the *ex vitro* leaves in the study. Nevertheless, neither methylation level SV or locus-specific MSV of *L*. *ruthenicum* showed a simple reduction after acclimatization because there was not only decrease but also increase; however, the decrease was predominant. On one hand, after acclimatization not only micropropagated plants but also donor plants of *L*. *ruthenicum* show significant DNA methylation changes. On the other hand, micropropagated plants and donors of *L*. *ruthenicum* show different response to acclimatization (S2 Table). Thus, some patterns of MSV, such as CG Hyper, increased significantly after acclimatization.

The SV is undesirable for long-term germplasm preservation [27] but can be used in strain improvement during plant breeding [25, 26]. The three types of transplanted micropropagated plants were diverged more from each other than the *in vitro* counterparts. Thus, it was difficult to select the most fidelity micropropagated plants *in vitro* but easier *ex vitro* (Figs 4 and 5). In conclusion, after acclimatization plants from stem calli were diverged more from the *ex vitro* donors than plants from axillary bud or leaf calli. Thus, we proposed that direct organogenesis from axillary buds might be suitable for preservation or propagation of elite *L*. *ruthenicum* germplasm. Meanwhile, the SV in plants from stem calli might be used to SV breeding. Also, the stem explants can be used in researching the novel sugar mechanism of dedifferentiation. However, propagation using *L*. *ruthenicum* leaf as explants can be used in germplasm preservation or propagation and is suitable for transformation. Furthermore, one MSV hotspot of *L*. *ruthenicum* was found based on 41 replicates of two groups. The hotspot indicated that certain regions of the *L*. *ruthenicum* genome are consistently exhibiting DNA demethylation in tissue culture. This is similar to recent studies in rice and maize which have shown that losses of DNA methylation following tissue culture are more common than gains of DNA methylation [28, 58]. The hotspot of *L*. *ruthenicum* could be used for revealing the epigenetic mechanism of SV.

## Conclusions

We developed a novel efficient micropropagation protocol from leaves and stems of *L*. *ruthenicum* and found that stem explant dedifferentiation and redifferentiation, multiple shoots formation from axillary bud, redifferentiation of leaf callus and rooting of *L*. *ruthenicum* were all affected by genetic background. Notably, the optimal medium for indirect organogenesis of stem explants was auxin-free medium with 4% sucrose. This indicated that sucrose supply might be the crucial regulator of stem callus induction and proliferation in *L*. *ruthenicum*. One MSV hotspot was found based on MSAP analysis, which offer an important clue for revealing the epigenetic mechanism of SV. Furthermore, MSAP analysis indicated that DNA methylation SV of CNG decreased but that of CG increased after acclimatization; the three types of micropropagated plants (from leaf calli, from stem calli and from axillary buds) were epigenetically diverged more from each other after acclimatization. Thus, we proposed that the *ex vitro* micropropagated plants should be selected to determine the fidelity. In summary, micropropagation from axillary buds and leaves of *L*. *ruthenicum* was more fidelity and might be suitable for preservation or propagation of elite germplasm. Propagation using *L*. *ruthenicum* leaf as explants is suitable for transformation. Meanwhile, the micropropagation from stem calli showed the highest MSV and could be used in both SV breeding and researching the novel sugar mechanism of dedifferentiation. The findings above not only provided foundations for molecular breeding, somaclonal variation breeding, preservation and propagation of germplasm, but also offer clues for further theoretical researches.

## Supporting information

S1 Table Primer pairs used for MSAP analysis. (DOC)

S2 Table Changes in cytosine methylation pattern in *ex vitro* plants compared with the corresponding *in vitro* plants of *L*. *ruthenicum*. (DOCX)

S3 Table *In vitro* micropropagated plant-specific MSAP sites of *L*. *ruthenicum* D group. (DOCX)

S4 Table *In vitro* micropropagated plant-specific MSAP sites of *L*. *ruthenicum* G group. (DOCX)
