# Supplementary material for: A novel micropropagation of Lycium ruthenicum and epigenetic fidelity assessment of three types of micropropagated plants in vitro and ex vitro
Source: PLoS One. 2021 Feb 23;16(2):e0247666. doi: 10.1371/journal.pone.0247666 (PMC7901770; doi:10.1371/journal.pone.0247666)
Supplement: S1 Table — (DOC) [file pone.0247666.s001.doc]

**S1 Table.** **Primer pairs used for MSAP analysis.**

| Primer pairs  (fluorescent dye labels) | EcoRI + 3 primers (5′ to 3′) | HpaII/MspI + 3 primers (5′ to 3′) |
| --- | --- | --- |
| A4 ((FAM) | GACTGCGTACCAATTCAAC | ATCATGAGTCCTGCTCGGTTC |
| B4 (TAMARA) | GACTGCGTACCAATTCAAG | ATCATGAGTCCTGCTCGGTTC |
| B5 (TAMARA) | GACTGCGTACCAATTCAAG | ATCATGAGTCCTGCTCGGTTG |
| D1 (HEX) | GACTGCGTACCAATTCACT | ATCATGAGTCCTGCTCGGTCT |
| D4 (HEX) | GACTGCGTACCAATTCACT | ATCATGAGTCCTGCTCGGTTC |
| D5 (HEX) | GACTGCGTACCAATTCACT | ATCATGAGTCCTGCTCGGTTG |
| F4 (HEX) | GACTGCGTACCAATTCACG | ATCATGAGTCCTGCTCGGTTC |
| F6 (HEX) | GACTGCGTACCAATTCACG | ATCATGAGTCCTGCTCGGTTA |
| G5 (FAM) | GACTGCGTACCAATTCAGC | ATCATGAGTCCTGCTCGGTTG |
| G8 (FAM) | GACTGCGTACCAATTCAGC | ATCATGAGTCCTGCTCGGTGT |
| H2 (FAM) | GACTGCGTACCAATTCAGG | ATCATGAGTCCTGCTCGGTCG |
| H4 (FAM) | GACTGCGTACCAATTCAGG | ATCATGAGTCCTGCTCGGTTC |
| H5 (FAM) | GACTGCGTACCAATTCAGG | ATCATGAGTCCTGCTCGGTTG |
| H6 (FAM) | GACTGCGTACCAATTCAGG | ATCATGAGTCCTGCTCGGTTA |
