# Supplementary material for: A novel micropropagation of Lycium ruthenicum and epigenetic fidelity assessment of three types of micropropagated plants in vitro and ex vitro
Source: PLoS One. 2021 Feb 23;16(2):e0247666. doi: 10.1371/journal.pone.0247666 (PMC7901770; doi:10.1371/journal.pone.0247666)
Supplement: S2 Table — (DOCX) [file pone.0247666.s002.docx]

**S2 Table. Changes in cytosine methylation pattern in *ex vitro* plants compared with the corresponding *in vitro* plants of *L. ruthenicum.***

| **Comparison of plants before and after acclimatization** | **Patterns [frequencies (%)]** | | | | | | | | |
| --- | --- | --- | --- | --- | --- | --- | --- | --- | --- |
|  | **CG Hyper** | **CG Hypo** | **CNG Hyper** | **CNG Hypo** | **Both Hyper** | **Both Hypo** | **Total Hyper** | **Total Hypo** | **Total** |
| *exDdonor* vs. *inDdonor* | 5.98 | 12.28 | 12.95 | 9.89 | 2.19 | 1.46 | 21.12 | 23.64 | 44.76 |
| *exGdonor* vs. *inGdonor* | 11.96 | 10.90 | 7.31 | 9.37 | 1.46 | 2.72 | 20.73 | 22.99 | 43.72 |
| Mean1 | 8.97 | 11.59^*^ | 10.13 | 9.63^ab*^ | 1.83 | 2.09 | 20.93^**^ | 23.32^**^ | 44.24^**^ |
| *exDaxil-plant_1_* vs. *inDaxil-plant_1_* | 8.08 | 9.90 | 6.67 | 10.67 | 0.77 | 2.60 | 15.52 | 23.17 | 38.69 |
| *exDaxil-plant_2_* vs. *inDaxil-plant_2_* | 8.57 | 9.65 | 4.61 | 9.43 | 1.51 | 1.80 | 14.69 | 20.88 | 35.57 |
| *exGaxil-plant_1_* vs. *inGaxil-plant_1_* | 8.88 | 10.16 | 8.08 | 10.63 | 1.62 | 1.28 | 18.57 | 22.07 | 40.64 |
| *exGaxil-plant_2_* vs. *inGaxil-plant_2_* | 7.99 | 10.55 | 9.37 | 10.42 | 2.95 | 2.69 | 20.31 | 23.66 | 43.97 |
| *exGaxil-plant_3_* vs. *inGaxil-plant_3_* | 7.24 | 9.08 | 9.16 | 8.87 | 1.28 | 1.49 | 17.67 | 19.45 | 37.12 |
| Mean2 | 8.15^**^ | 9.87^**^ | 7.58^**^ | 10.00^ab**^ | 1.63^*^ | 1.97^**^ | 17.35^**^ | 21.85^**^ | 39.20^**^ |
| *exDstem-plant_1_* vs. *inDstem-plant_1_* | 6.44 | 7.00 | 9.45 | 16.45 | 1.33 | 1.26 | 17.21 | 24.70 | 41.91 |
| *exDstem-plant_2_* vs. *inDstem-plant_2_* | 7.45 | 8.31 | 8.52 | 10.67 | 1.79 | 3.01 | 17.77 | 21.99 | 39.76 |
| *exDstem-plant_3_* vs. *inDstem-plant_3_* | 5.69 | 10.20 | 7.65 | 11.59 | 0.73 | 1.82 | 14.07 | 23.62 | 37.69 |
| *exDstem-plant_4_* vs. *inDstem-plant_4_* | 7.93 | 8.65 | 5.33 | 8.29 | 1.22 | 1.87 | 14.48 | 18.80 | 33.28 |
| *exGstem-plant_1_* vs. *inGstem-plant_1_* | 9.14 | 7.38 | 8.68 | 16.97 | 1.50 | 3.79 | 19.32 | 28.13 | 47.45 |
| *exGstem-plant_2_* vs. *inGstem-plant_2_* | 8.64 | 8.22 | 9.41 | 12.50 | 2.67 | 2.32 | 20.72 | 23.03 | 43.75 |
| *exGstem-plant_3_* vs. *inGstem-plant_3_* | 10.91 | 7.78 | 9.38 | 12.29 | 3.78 | 1.89 | 24.07 | 21.96 | 46.03 |
| *exGstem-plant_4_* vs. *inGstem-plant_4_* | 9.56 | 5.00 | 7.28 | 8.42 | 1.21 | 0.64 | 18.06 | 14.06 | 32.12 |
| Mean3 | 8.22^**^ | 7.82^**^ | 8.21^**^ | 12.15^a**^ | 1.78^**^ | 2.08^**^ | 18.21^**^ | 22.04^**^ | 40.25^**^ |
| *exDleaf-plant_1_* vs. *inDleaf-plant_1_* | 8.92 | 8.71 | 7.16 | 9.83 | 2.88 | 1.69 | 18.96 | 20.22 | 39.18 |
| *exDleaf-plant_2_* vs. *inDleaf-plant_2_* | 7.76 | 13.63 | 7.13 | 9.92 | 2.66 | 4.40 | 17.54 | 27.95 | 45.49 |
| *exDleaf-plant_3_* vs. *inDleaf-plant_3_* | 8.56 | 15.18 | 8.06 | 6.12 | 2.16 | 2.37 | 18.78 | 23.67 | 42.45 |
| *exDleaf-plant_4_* vs. *inDleaf-plant_4_* | 7.39 | 9.64 | 9.92 | 9.03 | 1.71 | 3.49 | 19.02 | 22.16 | 41.18 |
| *exGleaf-plant_1_* vs. *inGleaf-plant_1_* | 9.52 | 7.76 | 8.44 | 10.07 | 1.36 | 0.88 | 19.32 | 18.71 | 38.03 |
| *exGleaf-plant_2_* vs. *inGleaf-plant_2_* | 12.85 | 6.06 | 7.94 | 7.47 | 1.35 | 0.74 | 22.14 | 14.27 | 36.41 |
| *exGleaf-plant_3_* vs. *inGleaf-plant_3_* | 10.31 | 5.80 | 8.73 | 9.38 | 1.15 | 1.36 | 20.19 | 16.54 | 36.73 |
| *exGleaf-plant_4_* vs. *inGleaf-plant_4_* | 8.48 | 5.06 | 8.67 | 13.53 | 1.18 | 3.02 | 18.33 | 21.62 | 39.95 |
| Mean4 | 9.22^**^ | 8.98^**^ | 8.26^**^ | 9.42^b**^ | 1.81^**^ | 2.24^**^ | 19.29^**^ | 20.64^**^ | 39.93^**^ |

* Difference at 0.05 level by one-sample t-test, ** Difference at 0.01 level by one-sample t-test.

Data within columns labeled with different letters are significantly different at the 0.05 level by LSD.
