# Supplementary material for: A novel micropropagation of Lycium ruthenicum and epigenetic fidelity assessment of three types of micropropagated plants in vitro and ex vitro
Source: PLoS One. 2021 Feb 23;16(2):e0247666. doi: 10.1371/journal.pone.0247666 (PMC7901770; doi:10.1371/journal.pone.0247666)
Supplement: S3 Table — (DOCX) [file pone.0247666.s003.docx]

**S3 Table. *In vitro* micropropagated plant-specific MSAP sites of *L. ruthenicum* D group.**

| **Primer pairs** | | **A4** | **A4** | **A4** | **A4** | **A4** | **B4** | **G5** | **G5** | **G5** | **G5** | **G5** | **H4** | **H4** | **H4** |
| --- | --- | --- | --- | --- | --- | --- | --- | --- | --- | --- | --- | --- | --- | --- | --- |
| Length (bp) | | 52 | 68 | 102 | 103 | 146 | 140 | 189 | 190 | 202 | 203 | 204 | 153 | 207 | 239 |
| *inDdonor* | *Eco*RⅠ/ *Hpa*Ⅱ | 0 | 0 | 0 | 0 | 0 | 0 | 0 | 1 | 1 | 0 | 0 | 0 | 1 | 0 |
|  | *Eco*RⅠ/ *Msp*Ⅰ | 1 | 1 | 1 | 1 | 1 | 1 | 1 | 0 | 0 | 1 | 0 | 1 | 0 | 1 |
| *inDaxil-plants_1-2_*, *inDstem-plants_1-4,_ inDleaf-plants_1-4_* | *Eco*RⅠ/ *Hpa*Ⅱ | 0 | 0 | 0 | 0 | 0 | 1 | 1 | 0 | 1 | 0 | 1 | 1 | 1 | 0 |
|  | *Eco*RⅠ/ *Msp*Ⅰ | 0 | 0 | 0 | 0 | 0 | 1 | 1 | 0 | 1 | 0 | 0 | 1 | 1 | 0 |
