# Supplementary material for: A novel micropropagation of Lycium ruthenicum and epigenetic fidelity assessment of three types of micropropagated plants in vitro and ex vitro
Source: PLoS One. 2021 Feb 23;16(2):e0247666. doi: 10.1371/journal.pone.0247666 (PMC7901770; doi:10.1371/journal.pone.0247666)
Supplement: S4 Table — (DOCX) [file pone.0247666.s004.docx]

**S4 Table. *In vitro* micropropagated plant-specific MSAP sites of *L. ruthenicum* G groups.**

| **Primer pairs** | | **B4** | **G5** |
| --- | --- | --- | --- |
| Length (bp) | | 106 | 128 |
| *inGdonor* | *Eco*RⅠ/ *Hpa*Ⅱ | 0 | 0 |
|  | *Eco*RⅠ/ *Msp*Ⅰ | 1 | 1 |
| *inGaxil-plants_1-3_*, *inGstem-plants_1-4,_ inGleaf-plants_1-4_* | *Eco*RⅠ/ *Hpa*Ⅱ | 1 | 1 |
|  | *Eco*RⅠ/ *Msp*Ⅰ | 1 | 1 |
